# Cytotoxic and chemomodulatory effects of *Phyllanthus niruri* in MCF-7 and MCF-7^ADR^ breast cancer cells

**DOI:** 10.1038/s41598-023-29566-0

**Published:** 2023-02-15

**Authors:** Ola E. Abdel-Sattar, Rasha Mosa Allam, Ahmed M. Al-Abd, Bharathi Avula, Kumar Katragunta, Ikhlas A. Khan, Ahmed M. El-Desoky, Shanaz O. Mohamed, Ali El-Halawany, Essam Abdel-Sattar, Meselhy R. Meselhy

**Affiliations:** 1 Pharmacognosy Department, Faculty of Pharmacy, Cairo University, Kasr El Aini St., Cairo, 11562 Egypt; 2 Pharmacology Department, Medical and Clinical Research Institute, National Research Centre, Dokki, Cairo, 12622 Egypt; 3 Biomedical Research Division, NAWAH Scientific, Mokattam, Cairo, Egypt; 4 National Center for Natural Products Research, School of Pharmacy, University of Mississippi, University, MS 38677 USA; 5 Division of Pharmacognosy, Department of BioMolecular Sciences, School of Pharmacy, University of Mississippi, University, MS 38677 USA; 6 Department of Molecular Biology, Genetic Engineering and Biotechnology Research Institute (GEBRI), University of Sadat City (USC), Sadat City, 32958 Egypt; 7 School of Pharmaceutical Sciences, Universiti Sains Malaysia, 11700 Gelugor, Penang Malaysia

**Keywords:** Biochemistry, Biophysics, Cancer, Drug discovery, Plant sciences

## Abstract

The members of the genus *Phyllanthus* have long been used in the treatment of a broad spectrum of diseases. They exhibited antiproliferative activity against various human cancer cell lines. Breast cancer is the most diagnosed cancer and a leading cause of cancer death among women. Doxorubicin (DOX) is an anticancer agent used to treat breast cancer despite its significant cardiotoxicity along with resistance development. Therefore, this study was designed to assess the potential cytotoxicity of *P. niruri* extracts (and fractions) alone and in combination with DOX against naïve (MCF-7) and doxorubicin-resistant breast cancer cell lines (MCF-7^ADR^). The methylene chloride fraction (CH_2_Cl_2_) showed the most cytotoxic activity among all tested fractions. Interestingly, the CH_2_Cl_2_-fraction was more cytotoxic against MCF-7^ADR^ than MCF-7 at 100 µg/mL. At sub-cytotoxic concentrations, this fraction enhanced the cytotoxic effect of DOX against the both cell lines under investigation (IC_50_ values of 0.054 µg/mL and 0.14 µg/mL vs. 0.2 µg/mL for DOX alone against MCF-7) and (1.2 µg/mL and 0.23 µg/mL vs. 9.9 µg/mL for DOX alone against MCF-7^ADR^), respectively. Further, TLC fractionation showed that B2 subfraction in equitoxic combination with DOX exerted a powerful synergism (IC_50_ values of 0.03 µg/mL vs. 9.9 µg/mL for DOX alone) within MCF-7^ADR^. Untargeted metabolite profiling of the crude methanolic extract (MeOH) and CH_2_Cl_2_ fraction exhibiting potential cytotoxicity was conducted using liquid chromatography diode array detector-quadrupole time-of-flight mass spectrometry (LC-DAD-QTOF). Further studies are needed to separate the active compounds from the CH_2_Cl_2_ fraction and elucidate their mechanism(s) of action.

## Introduction

Several challenges for cancer treatment might result in treatment failure such as, acquired drug resistance which results in the decrease in the intracellular drug concentrations with limited cancer proliferation inhibition and ultimately metastasis^1^. Accordingly, different dose regimens, higher chemotherapeutic doses or prolonged treatment might be needed to achieve similar efficacy which will lead to more adverse effects^2^. Doxorubicin (DOX), is the standard first-line adjuvant chemotherapy in the treatment of solid tumors of disparate origins especially, early and metastatic breast cancer, one of the most common female malignant neoplasms^3,4^. Despite showing promising clinical results, there are clinical limitations arising from its vulnerability to the development of multidrug resistance (MDR), possibly due to the overexpression of ATP binding cassette transporters as well as developing debilitating side effects hurdle against dose escalation and completion of treatment course^5,6^. Cardiotoxicity and other multi-organ toxicities such as liver are the major limitation for doxorubicin treatment continuation and dose escalation and accordingly, result in restricted anticancer efficacy^7,8^.

Therefore, there is a necessity for a proper approach to potentiate the DOX chemotherapeutic efficacy and limit its side effects by reducing its doses. It is worth mentioning that cancer cells typically display much higher ROS levels which counteract the activity of several cytotoxic agents such as DOX^9^.

Yet, cancer cells are naturally more dependent on antioxidant systems and ROS scavenging metabolic pathways. Consequently, impairing antioxidant system may be a potential therapeutic approach against cancer^10^.

To this aim, natural products are gaining prominent attention in drug discovery, especially for the development of anticancer drugs. Several plant extracts and bioactive compounds demonstrated promising anticancer properties as well as chemomodulatory effects^11,12^.

Genus *Phyllanthus *is one of the largest genus in the family Phyllanthaceae having 11 sub-genera compromising over 700 well-known species and are globally disseminated in the tropics and subtropics^13^. *Phyllanthus niruri* L. (Syn *P. amarus* Schum & Thonn) is a traditional pharmaceutical plant that has been described for its various medicinal activities such as hepatoprotective, antibacterial, antiviral, anti-diabetes, anti-obesity, antihyperlipidemic, and anti-inflammatory^14–17^.

In recent years, *Phyllanthus* species are examples of promising sources of natural anticancer products^18,19^. *P. niruri* demonstrated anticancer properties against lung cancer^20^**,** breast cancer^21^, prostate cancer^22^, and hepatocellular carcinoma^23^ . Thanks to its antiproliferative effect on cancer cells by modulating various cell signaling pathways, reports showed its advantage of being safe on normal cells^23,24^. Yet, its efficacy against high recurrent and resistant cancer such as breast cancer is still unclear.

In our previous work, we prepared lignan-rich extract from the aerial parts of *P. niruri *using nonconventional methods to increase the level of lignans calculated as phyllanthin^25^. Herein, we investigated the potential cytotoxicity of the different extracts from *P. niruri* and studied the influence of their combination with DOX on the cytotoxic profile of DOX in naïve and doxorubicin-resistant breast cancer cell lines.

## Materials and methods

### Chemicals

Phyllanthin was purchased from Fluka (Lot # BCBL2476V, product of India). The analytical grade solvents used in the extraction and chromatographic separation were purchased from El Gomhouria for Drugs Co. (Cairo, Egypt). Precoated TLC plates (20 × 20 cm), silica gel F_254_ and RP silica gel were obtained from Sigma-Aldrich chemicals (Darmstadt, Germany). Sulforhodamine-B (SRB) and doxorubicin were purchased from Sigma Chemical Co. (St. Louis, MO, USA). Acetonitrile, methanol, and formic acid used (HPLC grade) were obtained from Fisher Scientific, Waltham, MA, USA. Water was purified using a Milli-Q system (Millipore, Bedford, MA, USA).

### Plant material

Samples of aerial parts of *P. niruri* L. were collected in August 2018, from Tasek Gelugor, Penang, Malaysia and the collection procedure was in compliance with the national and international guidelines and legislation. The plant materials were supplied and identified by the staff members of the Malaysian Agricultural Research and Development Institute (MARDI) through Natural Wellness Co., Cairo, Egypt. A voucher specimen (PN-01-082018B) was kept at the herbarium of the Faculty of Pharmacy, Cairo University.

### Extraction and fractionation of the crude extract

The powdered aerial parts of *P. niruri* (750 g) were exhaustively extracted with MeOH (3 × 1.5 L) and another amount (100 g) was extracted with boiling distilled water (2 × 150 mL) using homogenizer (3 times each). The solvent was removed under vacuum, at a temperature not exceeding 60 °C to yield 93 g of MeOH extract (**Ext-1**) and 16 g of water extract (**Ext-2**). Part of the MeOH extract (70 g) was suspended in H_2_O (600 mL) and extracted with CH_2_Cl_2_ (3 × 400 mL). The solvent was removed by distillation to yield CH_2_Cl_2_ fr. (**Fr-3**, 24.5 g). The aqueous remaining fraction (44 g) was passed through Diaion-HP20 (4 × 20 cm) and eluted with 100% water (500 mL), 50% MeOH (500 mL) followed with 100% MeOH (750 mL) to give **Fr-4** (26.2 g), **Fr-5 (**13.2 g), and **Fr-6** (4.1 g), respectively. The resulting MeOH extract and CH_2_Cl_2_ fraction were analyzed by Liquid chromatography diode array detector-quadrupole time-of-flight mass spectrometry (LC-DAD-QTOF) and were biologically tested.

The CH_2_Cl_2_ fr. (10% solution in CH_2_Cl_2_) was subjected to preparative TLC (solvent system: n-hexane-ethyl acetate, 2:1) on silica gel plates (Silica gel 60, Merck; 0.5 mm thick, 20 × 20 cm). Each plate was divided into 4 bands (**B1**–**B4**) and each band was scratched, eluted with a mixture of CH_2_Cl_2_-MeOH (1:1), and evaporated and the the samples kept at 4 °C till use.

### LC–MS analysis

#### Sample preparation

Samples from the MeOH extract and CH_2_Cl_2_ fraction were prepared in MeOH (LC–MS grade) in concentration of 50 mg/mL. The solution was then filtered and stored in sealed glass vials until analysis.

#### Liquid chromatography diode array detector-quadrupole time-of-flight mass spectrometry (LC-DAD-QToF)

The liquid chromatographic system was an Agilent Series 1290 and the separation was achieved on an Acquity UPLC™ HSS C18 column (100 mm × 2.1 mm I.D., 1.8 µm). The mobile phase consisted of water with 0.1% formic acid (A) and acetonitrile with 0.1% formic acid (B) at a flow rate of 0.21 mL/min. The analysis was performed using the following gradient elution: 95% A/5% B to 35%B in 15 min, in next 5 min to 70%B and finally to 100% B in next 2 min. A 3 min wash followed each run with 100% B and an equilibration period of 5 min with 95% A/5% B. One microliter of the sample was injected. The column temperature was 40 °C.

The mass spectrometric analysis was performed with a QTOF-MS–MS (Model #G6545B, Agilent Technologies, Santa Clara, CA, USA) equipped with an ESI source using the following parameters: drying gas (N_2_) flow rate, 13 L/min; drying gas temperature, 300 °C; nebulizer pressure, 30 psig, sheath gas temperature, 400 °C; sheath gas flow, 12 L/min; capillary voltage, 4000 V; nozzle voltage, 0 V; skimmer, 65 V; Oct RF V, 750 V; and fragmentor voltage, 150 V. All the operations, acquisition and analysis of data were controlled by Agilent MassHunter Acquisition Software Ver. A.10.1 and processed with MassHunter Qualitative Analysis software Ver. B.10.00. Each sample was analyzed in positive and negative modes over the range of *m/z* = 50–1100 and extended dynamic range (flight time to *m/z* 1700 at 2 GHz acquisition rate). Accurate mass measurements were obtained by means of reference ion correction using reference masses at *m/z* 121.0509 (protonated purine) and 922.0098 [protonated hexakis (1H, 1H, 3H-tetrafluoropropoxy) phosphazine or HP-921] in positive ion mode, while at *m/z* 112.9856 (deprotonated trifluoroacetic acid-TFA) and 1033.9881 (TFA adducted HP-921) were used in negative ion mode. Samples were analyzed in all-ion MS–MS mode, where experiment 1 was carried out with collision energy of zero and experiment two with a fixed collision energy of 45 eV.

### Cytotoxicity assay

#### Cell culture

Human breast cancer cell line (MCF-7), doxorubicin-resistant breast cancer cell line (MCF-7^ADR^), rat normal cardiomyocytes (H9C2) and mouse normal hepatocytes (BNL) were obtained from Nawah Scientific Inc. (Mokatam, Cairo, Egypt) and cultured in DMEM (Dulbecco’s modified eagle’s medium), Gibco, USA. Fetal bovine serum (FBS) at a concentration of 10% and 100 units/mL of penicillin/streptomycin (PS) were added to the culture media. The cells were incubated at 37 °C in a humid environment that contained 5% CO_2_.

#### Cell viability assay

The cell viability was determined following the method reported by Skehan et al.^26^. MCF-7, MCF7^ADR^, H9C2 and BNL cells were seeded in 96‐well plates, approximately 10^5^/well. After treatment of DOX and/or *P. niruri* fractions and subfractions with concentrations of 0.01, 0.1, 1, 10 and 100 ug/ml for 72 h. We replaced the media with 150 μL of 10% TCA (trichloroacetic acid) (Merck) for 1 h at 4 °C, followed by 5 timed washing with distilled water. After that, we added 70 μL of 0.4% w/v SRB solution (Sigma‐Aldrich) at room temperature for ten minutes in the dark. Cells were washed with 1% acetic acid (Merck) 3 times and allowed to a 24 h air-dry. Then, we added 150 μL of Tris Base (10 mM) (Merck) and the O.D. was determined at 540 nm by a microplate reader (FluoStar Omega, BMG Labtec, Ortenberg, Germany).

#### Measurement of intracellular GSH Level

MCF-7^ADR^ cells were treated with DOX and B2 alone or combined at their pre-determined IC_50_ values for 48 h. After treatment, cells were washed with PBS, trypsinized, centrifuged, and pellets were lysed using 0.5 ml of ice-cold lysis buffer (Cat No: FNN001; Invitrogen). The intracellular GSH levels were determined using the commercially available colorimetric kit to determine the GSH content (Biodiagnostics Company, Cairo, Egypt) according to the manufacturer’s instructions^27^. The total protein content in the cell lysate was determined using Bradford method for normalization.

### Data analysis

The dose–response curves of compounds were investigated applying the E_max_ model as previously described^28^. The combination index (CI) was calculated as previously described^29^. Drug interactions are classified as additive if CI is between 0.8 and 1.2, antagonistic if CI is > 1.2, and synergistic if CI is < 0.8. All data are presented as means ± SD (n = 3). Analysis of variance (ANOVA) with Tukey’s post-hoc test (*P* < 0.05) was applied using GraphPad Prism Software version 6.

## Results

### RP‐LC‐DAD‐ESI‐QTOF‐MS profiling of methanol extract and methylene chloride fraction

**Figure 1:**
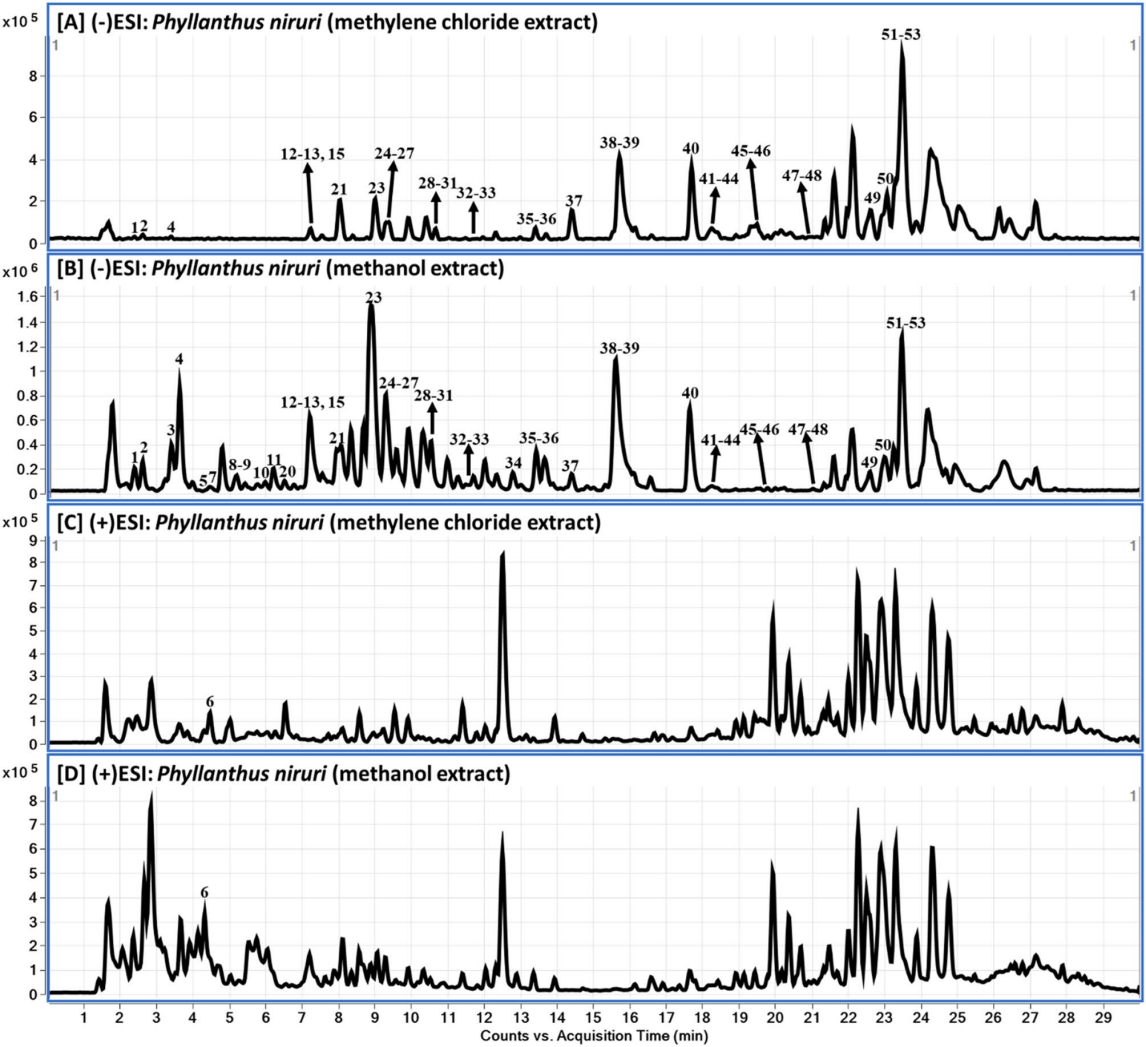
LC-QToF total Ion Chromatograms (TOC) of the CH_2_Cl_2_ and MeOH extract of *P. niruri*: in negative (**A**, **B**) and positive modes and (**C**, **D**).

**Figure 2:**
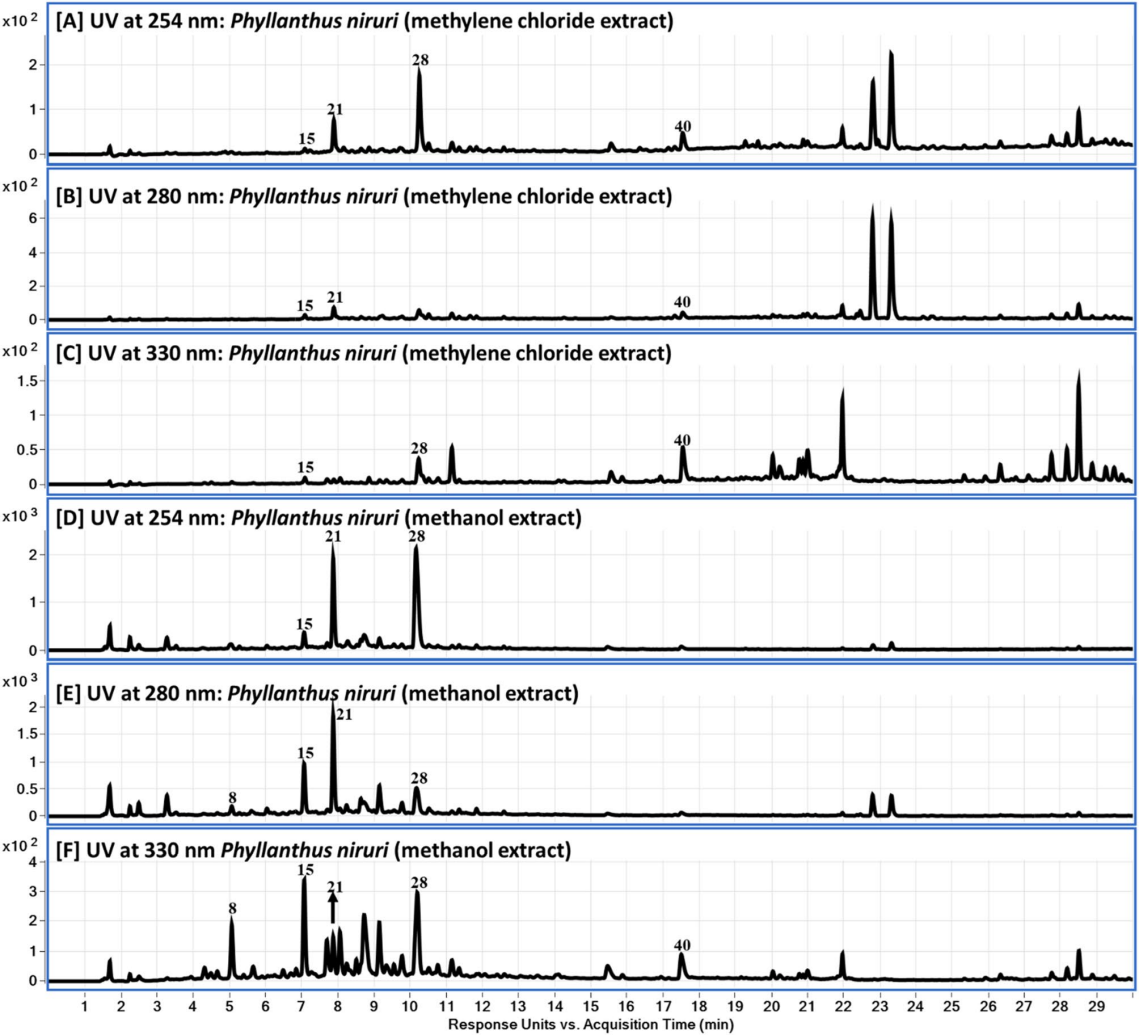
LC-DAD chromatograms of the CH_2_Cl_2_ fraction (**A**–**C**) and MeOH extract (**D**–**F**) of *P. niruri.* at 254, 280 and 330 nm.

The metabolite profiling of the methanol extract (MeOH) and the methylene chloride fraction (CH_2_Cl_2_) with potential cytotoxicity was analysed using liquid chromatography diode array detector-quadrupole time-of-flight mass spectrometry (LC-DAD-QToF). Figures 1 and 2 showed the base peak chromatograms (BPC) of the MeOH and the active CH_2_Cl_2_ fraction in negative and positive modes. Adopted analytical setup in both ESI modes led to the identification of 53 in both extracts with low abundance of metabolites **5**, **9–14**, within 30 min (Table 1). The metabolites were tentatively characterized based on their UV spectra (254, 280 and 330 nm), retention time, accurate mass, and MS fragmentation patterns and by comparing their mass spectra with those reported in the phytochemical dictionary of natural products database (CRC) and from the reported literature^30–33^.

**Table 1:** Tentative identification and characterization of phytochemical compounds in *Phyllanthus niruri* leaf and stem using LC-QToF in positive and negative ionization modes.

#	RT (min)	Compound Name	CH_2_Cl_2_	MeOH	Chemical class	Molecular Formula	Exact mass	[M + H]^+^	Fragment ions	[M–H]^−^	Fragment ions
1	2.4	2-Deoxy-2,3-dehydro-*N*-acetylneuraminic acid	**√**	**√**	Alpha-keto acid sugars	C_11_H_17_NO_8_	291.0954	–	–	290.0882 (290.0881)*	200.0567 [M–H–C_3_H_6_O_3_]^−^, 128.0356 [M–H–C_3_H_6_O_3_–C_3_H_4_O_2_]^−^
2	2.6	β-glucogallin 1-*O*-galloyl-B-D-glucopyranose,	**√**	**√**	Phenolic acid glycoside	C_13_H_16_O_10_	332.0743	–	–	331.0678 (331.0671)	211.0246 [M–H-C_4_H_8_O_4_]^−^, 169.0143 [M–H–C_6_H_10_O_5_]^−^, 125.0244 [M–H–C_6_H_10_O_5_–CO_2_]^−^
3	3.4	Gallic acid	**√**	**√**	Phenolic acid	C_7_H_6_O_5_	170.0215	171.0289 (171.0288)	–	169.0146 (169.0142)	125.0245 [M–H–CO_2_]^−^, 107.0139 [M–H–CO_2_–H_2_O]^−^
4	3.6	Vanillic acid 4-sulfate	**√**	**√**	Phenolic acid	C_8_H_8_O_7_S	247.9991	–	–	246.9922 (246.9918)	203.0019 [M–H–CO_2_]^−^, 121.0296 [M–H–CO_2_–SH_2_O_3_]^−^, 108.0220 [M–H–CO_2_–SH_2_O_3_–CH]^−^, 96.9604 [H_2_SO_4_]^−^, 80.9656 [H_2_SO_3_]^−^
5	4.07	Sesbanimide A	√*	**√**	Polyketide alkaloide	C_15_H_21_NO_7_	327.1318	328.1399 (328.1391)	310.1290 [M + H–H_2_O]^+^, 250.1655 [M + H–H_2_O–C_2_H_4_O_2_]^+^, 232.1550 [M + H–2H_2_O–C_2_H_4_O_2_]^+^, 132.0811 [M + H–2H_2_O–C_2_H_4_O_2_–C_4_H_4_O_3_]^+^, 120.0810 [M + H–2H_2_O–C_2_H_4_O_2_–C_5_H_4_O_3_]^+^	326.1243 (326.1245)	–
6	4.30	Phenylalanine	**√**	**√**	Amino acid	C_9_H_11_NO_2_	165.0790	166.0855 (166.0863)	120.0479	164.0719 (164.0717)	–
7	4.44	Caffeoylglucaric acid	**√**	**√**	Phenolic acid	C_15_H_16_O_11_	372.0693	–	–	371.0619 (371.0620)	315.0720 [M–H–C_2_O_2_]^−^, 209.0306 [M–H–C_2_O_2_–C_7_H_6_O]^−^
8	5.2	Dihydrocaffeic acid 3-*O*-glucuronide	**√**	**√**	Phenolic acid glycoside	C_15_H_18_O_10_	358.0900	–	–	357.0826 (357.0827)	269.0336, 219.0512, 151.0402, 123.0451
9	5.3	Protocatechuic acid	√*	**√**	Phenolic acid	C_7_H_6_O_4_	154.0266	–	–	153.0194 (153.0193)	–
10	5.75	Dihydro virganin	√*	**√**	phenolic	C_26_H_24_O_18_	624.0963	–	–	623.0883 (623.0890)	355.0665 [M–H–C_9_H_8_O_8_]^−^, 234.0405 [M–H–C_9_H_8_O_8_–C_7_H_5_O_2_]^−^
11	6.3	1,6-Digalloylglucose; D-pyranose-form	√*	**√**	Phenolic glycoside	C_20_H_20_O_14_	484.0853	–	–	483.0776 (483.0780)	169.0141, 125.0246
12	7.1	Phyllanthusiin B/ Phyllanthusiin G/ Phyllanemblinin C	√*	**√**	ellagitannins	C_41_H_30_O_28_	970.0924	–	–	969.0850 (969.0851)	925.0955
13	7.7		√*	**√**							
14	9.1		√*	**√**							
15	7.2	Brevifolin carboxylic acid	**√**	**√**	Phenol carboxylic acid	C_13_H_8_O_8_	292.0219	–	–	291.0157 (291.0146)	247.0250 [M–H–CO_2_]^−^, 219.0294 [M–H–CO_2_–CO]^−^, 191.0344 [M–H–CO_2_–2CO]^−^, 145.0292, 119.0505
16	7.56	Methyl gallate	**√**	**√**	Phenolic acid ester	C_8_H_8_O_5_	184.0372	–	–	183.0298 (183.0299)	124.0167 [M–H–CH_3_–CO_2_]^−^
17	7.8	Geraniin /Phyllanthusiin A/Geraniinic acid B	**√**	**√**	ellagitannin	C_41_H_28_O_27_	952.0818	–	–	951.0740 (951.0745)	–
18	9.8		**√**	**√**							
19	12.2		**√**	**√**							
20	6.9	Corilagin/1-*O*-Galloyl-6-*O*-luteoyl-α-D-glucopyranose	**√**	**√**	ellagitannins glycoside	C_27_H_22_O_18_	634.0806	652.1146 (652.1144) [M + NH_4_]^+^	465.0666 [M + H–C_7_H_6_O_5_]^+^,	633.0743 (633.0733)	463.0517 [M–H–C_7_H_6_O_5_]^−^, 300.9995, 275.0202
21	8.1		**√**	**√**							
22	8.8	Phyllanthusiin C	**√**	**√**	ellagitannin	C_40_H_30_O_26_	926.1025	–	–	925.0950 (925.0953)	511.0559
23	8.84	3,5,7-Trihydroxyflavone-8-sulfonic acid; 3-*O*-β-D-Glucopyranoside (Galangin-8-sulfonic acid, glucopyranoside	**√**	**√**	Flavnoid = flavone	C_21_H_20_O_13_S	512.0625	–	–	511.0555 (511.0552)	349.0026 [M–H–C_6_H_10_O_5_]^−^, 319.9996 [M–H–C_6_H_10_O_5_–CHO]^−^, 290.9974 [M–H–C_6_H_10_O_5_–2CHO]^−^, 269.0457 (galangin)
24	9.29	Brevifolin	**√**	**√**	Phenolic	C_12_H_8_O_6_	248.0015	–	–	247.0252 (247.0248)	219.0295, 191.0345, 145.0290, 117.0342
25	9.5	Phyllanemblinin A	**√**	**√**	Ellagitannins	C_27_H_20_O_17_	616.0700	–	–	615.0622 (615.0628)	300.9994, 169.0145
26	9.7	Quercetin-xylosyl-glucoside	**√**	**√**	Flavnoid = flavonol	C_26_H_28_O_16_	596.1377	597.1454 (597.1450)	303.0493	595.1299 (595.1305)	300.0272
27	9.90	Methylbrevifolin carboxylate	**√**	**√**	Phenolic	C_14_H_10_O_8_	306.0376	307.0453 (307.0448)	183.0921	305.0306 (305.0303)	273.0043 [M–H–CH_3_OH]^−^, 245.0085 [M–H–CH_3_OH–CO]^−^, 217.0136 [M–H–CH_3_OH–2CO]^−^, 201.0186 [M–H–CH_3_OH–CO–CO_2_]^−^, 189.0189 [M–H–CH_3_OH–3CO]^−^, 173.0237 [M–H–CH_3_OH–2CO–CO_2_]^−^,
28	10.30	Ellagic acid	**√**	**√**	Phenolic acid	C_14_H_6_O_8_	302.0063	–	–	300.9999 (00.9990)	283.9958 [M–H–OH]^−^, 257.0072 [M–H–CO_2_]^−^,
29	10.35	Quercetin-3-*O*-rutinoside	**√**	**√**	Flavnoid = flavonol	C_27_H_30_O_16_	610.1534	611.1611 (611.1607)	303.0501	609.1462 (609.1461)	301.0351
30	10.6	4-*O*-Brevifolincarbonyl-1-*O*-galloyl-3,6-*O*-hexahydroxydiphenoyl-D-glucopyranose	**√**	**√**	Phenolic	C_40_H_28_O_25_	908.0920			907.0847 (907.0844)	611.0708, 169.0141
31	10.88	Quercetin-*O*-hexoside	**√**	**√**	Flavnoid = flavonol	C_21_H_20_O_12_	464.0955	–	–	463.0877 (463.0877)	300.0257 [M–H–Hex]^−^, 271.0252 [M–Hex–CH_2_O]^−^, 255.0299 [M–Hex–CO–H_2_O]^−^, 151.0036
32	11.5	kaempferol 3-*O*-rutinoside	**√**	**√**	Flavnoid = flavonol	C_27_H_30_O_15_	594.1585	595.1661 (595.1657)	287.0556	593.1520 (593.1512)	285.0413
33	11.96	Chebulinic acid	**√**	**√**	Ellagitannins	C_41_H_32_O_27_	956.1131	–	–	955.1048 (955.1058)	923.0789 [M–H–CH_4_O]^−^, 879.0899 [M–H–CH_4_O–CO_2_]^−^, 611.0708 [M–H–CH_4_O–CO_2_–C_11_H_8_O_8_]^−^, 351.0176 [M–H–CH_4_O–CO_2_–C_11_H_8_O_8_–C_10_H_12_O_8_]^−^, 300.9988 [ellagic acid]^−^
34	12.72	Phyllanthussiin U	**√**	**√**	Ellagitannins	C_40_H_28_O_26_	924.0869	–	–	923.0792 (923.0796)	461.0359, 300.9988 [ellagic acid]
35	13.4	Azelaic acid	**√**	**√**	Dicarboxylic acid	C_9_H_16_O_4_	188.1049	–	–	187.0974 (187.0976)	–
36	13.7	3,4,5,7-Tetrahydroxyflavone-8-sulfonic acid	**√**	**√**	Flavnoid = flavone	C_15_H_10_O_9_S	366.0046	–	–	364.9973 (364.9973)	285.0403 [M–H–SO_3_]^−^
37	14.43	4-Hydroxybenzoic acid	**√**	**√**	Phenolic acid	C_7_H_6_O_3_	138.0317	–	–	137.0245 (137.0244)	93.0350 [M–H–CO_2_]^−^, 75.0243 [M–H–CO_2_–H_2_O]^−^
38	15.65	3,5,7-Trihydroxyflavone-8-sulfonic acid (Galangin-8-sulfonic acid)	**√**	**√**	Flavnoid = flavone	C_15_H_10_O_8_S	350.0096	–	–	349.0032 (349.0024)	269.0456 (galangin)
39	15.97		**√**	**√**							
40	17.7	Niruriflavone	**√**	**√**	Flavnoid = flavone	C_16_H_12_O_8_S	364.0253	–	–	363.0187 (363.0180)	347.9944, 268.0376, 151.0035
41	18.21	Trihydroxy-octadecadienoic acid	**√**	**√**	Fatty acid	C_18_H_32_O_5_	328.2250	–	–	327.2175 (327.2177)	285.0797, 215.1289, 171.1027, 85.0299
42	18.40		**√**	**√**							
43	18.60		**√**	**√**							
44	18.90	Trihydroxy-octadecanoic acid	**√**	**√**	Fatty acid	C_18_H_34_O_5_	330.2406	–	–	329.2332 (329.2333)	
45	19.75		**√**	**√**							
46	19.98		**√**	**√**							
47	21.0		**√**	**√**							
48	21.4	Nirtetralin	**√**	**√**	Lignan	C_24_H_30_O_7_	430.1992	431.2054 (431.2064)	–	–	–
49	22.7	Phyltetralin	**√**	**√**	Lignan	C_24_H_32_O_6_	416.2199	417.2278 (417.2272)	151.0755	–	–
50	22.9	Phyllanthin	**√**	**√**	Lignan	C_24_H_34_O_6_	418.2355	419.2428 **436.2694** (**436.2691**) 441.2248 (441.2249)	184.0740, 151.0764	–	–
51	23.4	Hypophyllanthin	**√**	**√**	Lignan	C_24_H_30_O_7_	430.1992	431.2065 (431.2064)	–	–	–
52	23.5	Niranthin	**√**	**√**	Lignan	C_24_H_32_O_7_	432.2148	433.2221 455.2045 (455.2040)	–	–	–
53	23.6	Isolintetralin	**√**	**√**	Lignan	C_23_H_28_O_6_	400.1886	401.1961 (401.1958)	151.0751	–	–

Significant values are in bold.

*Theoretical accurate mass; Compound #**5, 9–14** are present in low abundances in methylene chloride extracts.

**Figure 3:**
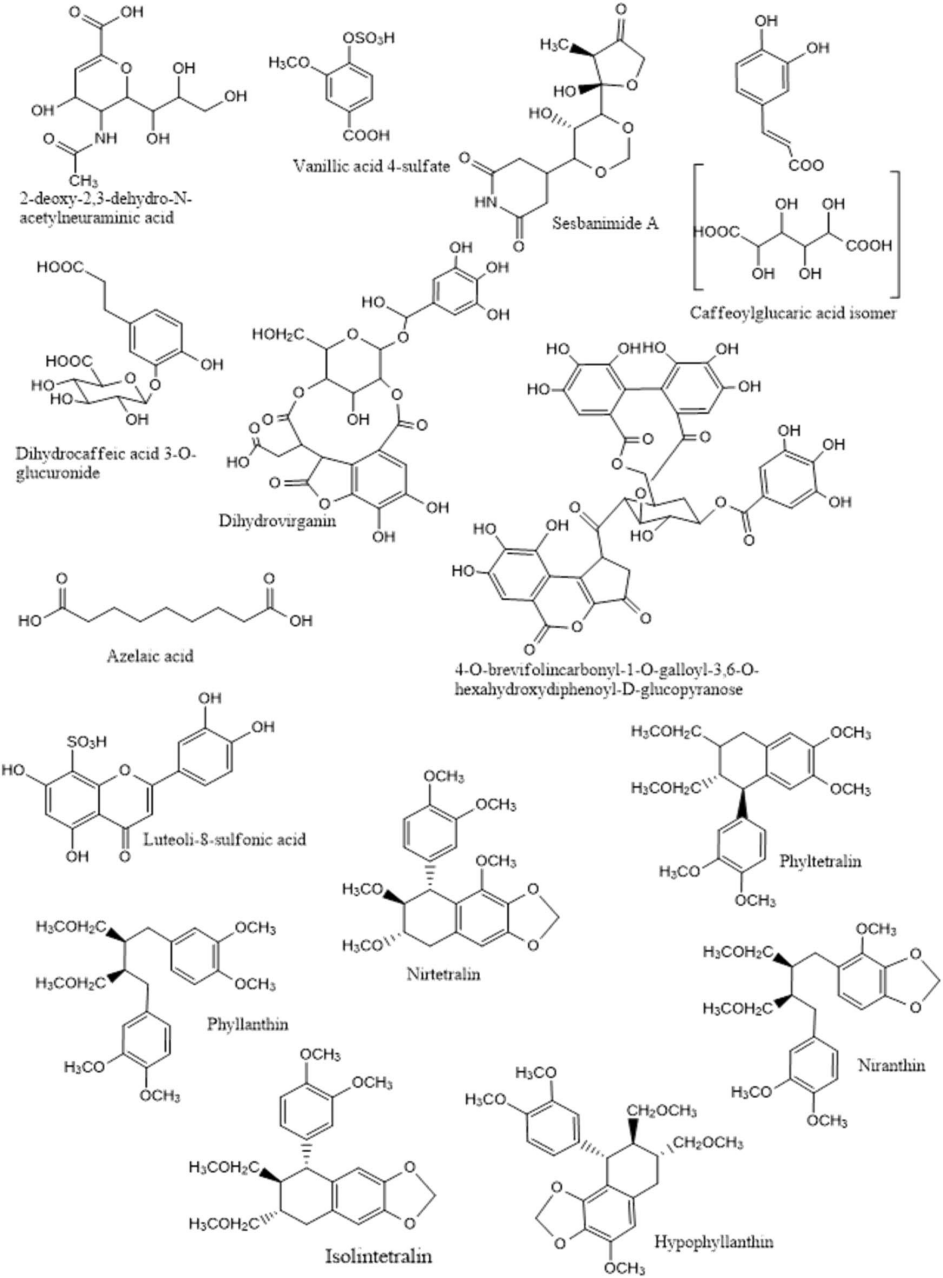
Examples of structure of some compound identified in *P. nirri.*

The identified compounds are represented by 42 phenolic acids and phenolic derivatives, 7 fatty acids, an amino acid, a sugar derivative, an alkaloid, and a dicarboxylic (Fig. 3 and Table 1). Most of peaks belonging to acids and phenolics were observed in negative ion mode (Table 1) showing [M–H]^−^ ions with the loss of one mass unit, except for compound **5** which was observed in positive mode. Details of metabolites assignment will be discussed in the coming section.

### Biological screening

#### Cytotoxicity assessment of *P. niruri*

Sulforhodamine B (SRB) assay was used to assess the potential cytotoxicity of six extracts of *P. niruri*, including total methanol (**Ext-1**), aqueous (**Ext-2**), CH_2_Cl_2_ (**Fr-3**), and diaion fractions (**Fr-4**: water, **Fr-5**: 50% MeOH and **Fr-6**: 100% MeOH), against naïve breast cancer cell line (MCF-7) and doxorubicin-resistant breast cancer cell line (MCF-7^ADR^).

All the tested fractions except for **Fr-3** did not show good cytotoxicity against the two cell lines since their IC_50_ values were higher than 100 μg/mL (Table 2). On the other hand, **Fr-3** showed substantial cytotoxicity against MCF-7 and MCF-7^ADR^ cells with IC_50_ values of 97.2 ± 1.13 μg/mL and 51.1 ± 0.93 μg/mL, respectively. Interestingly, MCF-7^ADR^ cells (Dox-resistant) were more sensitive than MCF-7 cells (Naïve) to Fr-3 treatment (Table 2).

**Table 2:** Cytotoxic activity of *P. niruri* of various extracts and fractions against MCF-7 and MCF-7^ADR^ cell lines.

Extract or fraction	MCF-7		MCF-7^ADR^	
	IC_50_ (µg/mL)	R-fraction (%)	IC_50_ (µg/mL)	R-fraction (%)
Ext-1	> 100	N/A	> 100	N/A
Ext-2	> 100	N/A	> 100	N/A
**Fr-3**	97.2 ± 1.13	N/A	51.1 ± 0.93	15.8 ± 0.7
Fr-4	> 100	N/A	> 100	N/A
Fr-5	> 100	N/A	> 100	N/A
Fr-6	> 100	N/A	> 100	N/A

^**#**^N/A means not applicable.

#### Chemomodulatory effect of *P. niruri* to doxorubicin (DOX) within naïve and resistant breast cancer cells

To study the outcome of *P. niruri* on the cytotoxic profile of DOX in MCF-7 and MCF-7^ADR^ cells, we assessed the dose–response curves of DOX alone and in combination with the different extracts of *P. niruri* (Fig. 1S-A, B) (Table 3).

**Table 3:** Chemomodulatory effects of *P. niruri* extracts and fractions on the cytotoxicity of DOX against MCF-7 and MCF-7^ADR^ cell lines**.**

Extract or fraction	MCF-7		MCF-7^ADR^		
	(IC_50_ µg/mL)	R-fraction (%)	(IC_50_ µg/mL)		R-fraction (%)
DOX	0.2 ± 0.06	0.44 ± 0.03	9.9 ± 1.1		5.2 ± 0.04
DOX + Ext-1 (100 µg/mL)	1.2 ± 0.08*	1.7 ± 0.3	0.27 ± 0.01*		N/A
DOX + Ext-2 (100 µg/mL)	1.9 ± 0.12*	N/A	38 ± 2.3*		N/A
DOX + Fr-3 (10 µg/mL)	0.054 ± 0.001*	N/A	0.23 ± 0.01*		N/A
DOX + Fr-4 (100 µg/mL)	1.6 ± 0.13*	N/A	9.9 ± 0.82		N/A
DOX + Fr-5 (100 µg/mL)	1.2 ± 0.1*	N/A	1.9 ± 0.45*		0.73 ± 0.01
DOX + Fr-6 (100 µg/mL)	0.8 ± 0.02*	1.9 ± 0.08	3.3 ± 0.07*		N/A

^**#**^N/A means not applicable. Data are presented as mean ± SD; n = 3.

*Significantly different from DOX treatment.

The chemomodulatory effect of *P. niruri* extracts and fractions at noncytotoxic concentrations were studied along with serial concentrations of DOX within breast cancer cells using SRB assay as described in the previous section.

In MCF-7 cells, DOX exerted a dose-dependent cytotoxic activity, and the viability started to decrease markedly at a concentration of 0.1 µg/mL with an IC_50_ value of 0.2 ± 0.06 µg/mL (Fig. 1S-A). Except for **Fr-3**, all tested extracts, and fractions of *P. niruri* (**Ext-1, Ext-2, Fr-4, Fr-5,** and** Fr-6**) at a sub-toxic concentration (100 µg/mL) in combination with DOX did not improve the cytotoxic/antiproliferative effect of DOX against MCF-7 cells. This can be observed from the increase of IC_50_ values of DOX to ~ 10 times after combination with the different extracts. Conversely, the combination of DOX with a sub-cytotoxic concentration of **Fr-3** (10 µg/mL), significantly increased the antiproliferative/cytotoxic effect of DOX against MCF-7 cells; the IC_50_ values of DOX were 0.2 ± 0.06 µg/mL and 0.054 ± 0.001 alone and in combination with **Fr-3**, respectively (Fig. 1S-A). A similar result was observed for the combination of DOX with **Fr-3** at higher concentration (100 µg/mL) and IC_50_ value were decreased to 0.014 ± 0.002 µg/mL (1S-A).

Similarly, in MCF-7^ADR^ cells, DOX also showed gradient cytotoxic activity with increasing concentration; viability started to drop significantly at a concentration of 1 μg/ml with an IC_50_ value of 9.9 ± 1.1 µg/mL (Fig. 1S-B). Unlike the MCF-7 cell line, combining **Ext**-**1, Fr-5,** and **Fr-6 **with DOX significantly improved the cytotoxic profile of DOX in MCF-7^ADR^ cells, decreasing both the resistant fraction and IC_50_ of DOX to 0.27 ± 0.01 μg/mL, 1.9 ± 0.45 μg/mL and 3.3** ± **0.07 μg/mL, respectively. Additionally, a more improvement in the cytotoxic profile of DOX was observed after the combination with **Fr-3** (10 μg/mL), decreasing IC_50_ of DOX to 0.23 ± 0.01 μg/mL along with abolishing the resistant fraction. On the other hand, the combination of **Fr-4** with DOX did not significantly influence the cytotoxic/antiproliferative activity of DOX against MCF-7^ADR^ cells.

The results of these two cell lines can declare that **Fr-3** possessed the most promising chemomodulatory effect for DOX in breast cancer cells. Therefore, additional subfractions from **Fr-3** were prepared and assessed for further cytotoxic and chemomodulatory potential.

#### Cytotoxicity and chemomodulatory assessment of different subfractions (B1–B4) of Fr-3 on MCF-7 and MCF-7^ADR^ cells

Four TLC subfractions from **fr-3** (CH_2_Cl_2_) were obtained by preparative TLC and named **B1, B2, B3**, and **B4**. In MCF-7cells, all the tested subfractions didn’t show observable cell-killing effects even until 100 μg/mL concentration. Conversely, in MCF-7^ADR^ cells, all subfractions showed cell-killing effects at a concentration of 100 μg/mL with calculated IC_50_ values of 188.6 ± 2.3 μg/mL, 86.4 ± 1.5 μg/mL, 100.4 ± 1.8 μg/mL and 112.7 ± 2 μg/ml for **B1, B2, B3** and **B4** respectively (Table 4). It is worth mentioning that MCF-7^ADR^ cells were more sensitive to treatment with these subfractions. Accordingly, it was selected to further investigate the chemomodulatory effect of these subfractions on DOX. Combinations of the four subfractions (**B1**–**4**) with DOX were clearly synergistic with very low CI-values of 0.157, 0.003, 0.038, 0.061, respectively (Table 5). Based on these CI-values, **B2** was the most promising fraction that significantly reduced the IC_50_ of DOX from 9.9 ± 1.1 to 0.03 ± 0.001 and abolished the resistance fraction from 5.2 ± 0.04 to nearly zero (Table 5). It merits mentioning that subfractions combinations with DOX drastically improved the cytotoxic profile of DOX against MCF-7^ADR^ (Fig. 2S).

**Table 4:** Cytotoxic effect of subfractions (B1–B4) of Fr-3 within MCF-7 and MCF7-^ADR^ cell lines.

Subfraction	MCF-7		MCF-7^ADR^	
	IC_50_ (μg/ml)	R-fraction (%)	IC_50_ (μg/ml)	R-fraction (%)
B1	> 100	N/A	188.6 ± 2.3	N/A
B2	> 100	N/A	86.4 ± 1.5	N/A
B3	> 100	N/A	100.4 ± 1.8	N/A
B4	> 100	N/A	112.7 ± 2	N/A

^**#**^*N/A* means not applicable.

**Table 5:** Chemomodulatory effect of subfractions (B1–B4) of Fr-3 on the cytotoxicity of DOX against MCF-7^ADR^ cell line.

Subfraction	MCF-7_ADR_	
	(IC_50_ µg/mL)	R-fraction (%)
**DOX**	9.9 ± 1.1	5.2 ± 0.04
DOX + **B1** (1:10)	1.5 ± 0.3*	N/A
CI index/ CI value	Synergism/0.157	
DOX + **B2** (1:10)	0.03 ± 0.001*	N/A
CI index/ CI value	Synergism/0.003	
DOX + **B3** (1:10)	0.35 ± 0.02*	N/A
CI index/ CI value	Synergism/0.038	
DOX + **B4** (1:10)	0.56 ± 0.04*	N/A
CI index/ CI value	Synergism/0.061	

^**#**^N/A means not applicable. Data are presented as mean ± SD; n = 3.

*Significantly different from DOX treatment.

#### Safety assessment of B2-fraction on H9C2 and BNL cells

The most promising cytotoxic fraction (B2) was further assessed for its possible cardiotoxic and/or hepatotoxic effects. H9C2 (normal cardiomyocyte) and BNL (normal hepatocytes) were incubated with B2 for 72 h, and cell viability was assessed by SRB and compared with the cell viability of DOX (Fig. 3S).

Dox inhibited cell proliferation of both H9C2 and BNL cells in a dose-dependent manner, with IC_50_ values of 0.0153 ± 0.001 µg/ml and 0.0571 ± 0.002 µg/ml, respectively. While B2-fraction did not affect the cell viability of both H9C2 and BNL cells up to a concentration of 100 µg/ml, indicating the safety of B2 fraction on heart and liver normal cells in contrast to DOX (Fig. 3S).

#### The effect of DOX, B2-fraction, and their combination on the intracellular GSH level within MCF-7^ADR^ cells

**Figure 4:**
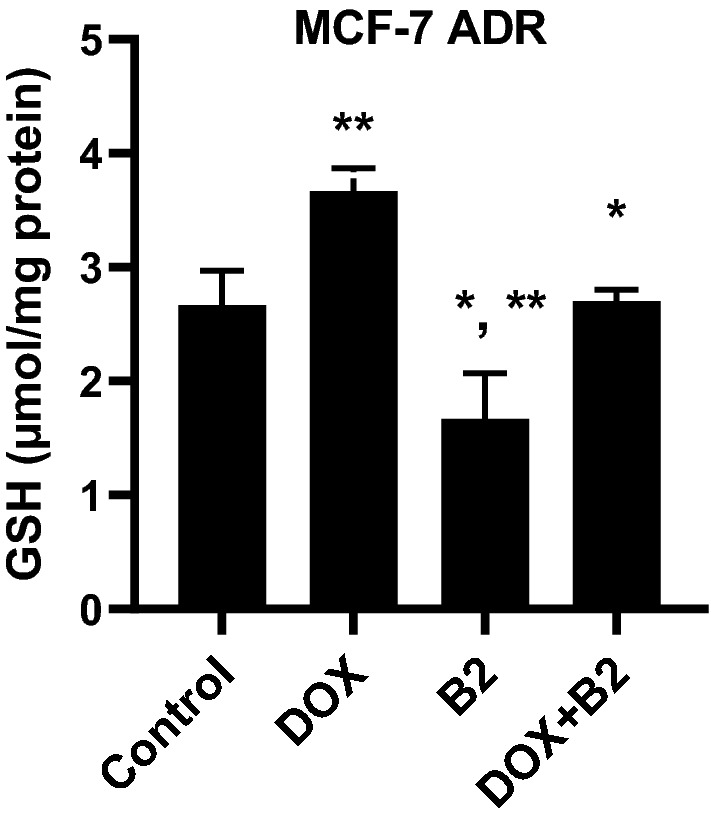
Effects of treatment with IC_50_ values of B2-fraction, DOX alone or their combination on the intracellular level of GSH within MCF-7^ADR^ cells. Data are presented as mean ± SD; n = 3. *Significantly different from DOX treatment. **Significantly different from control.

In this study, we tested the influence of the most potent cytotoxic fraction (B2) and doxorubicin alone or in combination on the intracellular level of GSH within MCF-7^ADR^ cells. Numerous studies have indicated GSH activity alterations after treatment with doxorubicin in vitro. Herein, it was found that DOX treatment resulted in a significant increase in GSH levels (3.81 ± 0.2 µmol/mg protein) compared to non-treated cells (2.71 ± 0.1 µmol/mg protein). On the other hand, B2 alone showed a significant reduction in GSH level to 1.67 ± 0.5 µmol/mg protein compared to non-treated cells (2.71 ± 0.1 µmol/mg protein). The combination of B2-fraction and DOX normalized the intracellular GSH level and brought back to be non-significant from untreated cells (Fig. 4).

## Discussion

### RP‐LC‐DAD‐ESI‐QTOF‐MS profiling of methanol extract and methylene chloride fraction

Untargeted metabolite profiling of the crude MeOH extract and the most active CH_2_Cl_2_ fraction with potential cytotoxicity was conducted using RP‐LC‐DAD‐ESI‐QTOF‐MS. The results of the analysis revealed the identification of 53 in both extracts with different abundance using both ESI modes (Table 1). The metabolites were tentatively characterized based on their UV spectra (254, 280 and 330 nm), retention time, molecular weight, MS fragmentation patterns and comparing their mass spectra with those reported in the phytochemical dictionary of natural products database (CRC) and reported literature^30–33^.

The identified metabolites (Fig. 3, Table 1) are belonging to different phytochemical classes, such as phenolic acids and phenolic derivatives (**14**), ellagitannins (**12**), flavonoids (**10**), fatty acids (**7**), lignans (**6**), and amino acid (**1**), sugar derivative (**1**), alkaloid (**1**), and dicarboxylic (**1**). Details of metabolites assignment will be discussed in the coming section.

Phenolic, phenolic acids, alkaloid, ellagitannins and flavonoid glycosides are eluted first in the chromatogram followed by fatty acids, flavonoid aglycones, while the lignans, and were emerged by end of the chromatogram. Most of peaks belonging to acids and phenolics compounds were observed in negative ion mode (Table 1).

Among the fifty-three identified metabolites, sixteen were reported in *P. niruri* for the first time and the metabolites identified were **1**, **4**, **5**, **7**, **8**, **10**, **30, 35, 36**, **41–47***.* The rest of the metabolites are either previously identified in *P. niruri* or from other *Phyllanthus* species. The newly identified metabolites in *P. niruri* and in genus *Phyllanthus* are classified into sialic acid derivative (**1**), phenolics and phenolic acid derivatives (**4, 7, 8, 10, and 30**), organic acid (**35**), a flavonoid (**36**), polyketide alkaloid (**5**), and fatty acids (**41–47**).

Compound **1** was identified as 2-deoxy-2,3-dehydro-*N*-acetylneuraminic acid (sialic acid derivative) by observing mass ions at 290.0882 [M-H]^-^, and fragments at *m/z* 200.0567 [M-H-C_3_H_6_O_3_]^-^, 128.0356 [M-H-C_3_H_6_O_3_-C_3_H_4_O_2_]^-^. This compound was previously identified in *Nymphaea nouchali* stem^34^, and detected in edible bird’s nest, a nutrient-rich salivary bioproduct produced by swiftlets in Southeast Asia^35^.

Compound **4** was identified as vanillic acid 4-sulfate, previously isolated from seaweeds *Sargassum* sp., *Centroceras* sp., *Ulva* sp.^36^, and detected in the urine of healthy rats supplemented for 35 days with cranberry extract^37^. It showed a molecular ion peak [M–H]^−^ at *m/z* 290.0882, in addition to the fragmentation pattern with ions at *m/z* 203.0019 [M–H–CO_2_]^−^, 121.0296 [M–H–CO_2_–SH_2_O_3_]^−^, 108.0220 [M–H–CO_2_–SH_2_O_3_–CH]^−^, 96.9604 [H_2_SO_4_]^−^, 80.9656 [H_2_SO_3_]^−^^38^.

Compound **5** was identified as sesbanimide A based on the fragmentation pattern with ions at *m/z* 328.1399 [M + H]^+^, 310.1290 [M + H–H_2_O]^+^, 250.1655 [M + H–H_2_O–C_2_H_4_O_2_]^+^, 232.1550 [M + H–2H_2_O–C_2_H_4_O_2_]^+^, 132.0811 [M + H–2H_2_O–C_2_H_4_O_2_–C_4_H_4_O_3_]^+^, 120.0810 [M + H–2H_2_O–C_2_H_4_O_2_–C_5_H_4_O_3_]^+^. This compound was detected and isolated from two marine alphaproteobacteria *Stappia indica* PHM037 and *Labrenzia aggregata* PHM038^39^, in Wu-Ling-San traditional Chinese formula^40^, and from *Sesbania drummondii* seed^41^.

Compounds **7** and **8** had deprotonated molecular ions [M–H]^−^ at *m/z* 371.0619 and 357.0826, and molecular formulae C_15_H_16_O_11_ and C_15_H_18_O_10_, respectively. They were tentatively identified as caffeoylglucaric acid isomer (**7**) isolated from edible wild calafate berry (*Berberis microphylla* G. Forst)^42^ and dihydrocaffeic acid 3-*O*-glucuronide (**8**) detected among the polyphenol-derived metabolites in human plasma after coffee consumption^43–45^. The identification of compound **7** as caffeoylglucaric acid isomer was achieved through the presence of the fragment ions at *m/z* 315, 209 and 191. According the reported literature^42^, the fragment at *m/z* 209 can be interpreted as an aldaric acid moiety like glucaric, with subsequent loss of H_2_O, to give rise the fragment ion at *m/z* 191 [209 − H_2_O]^−^. This compound was tentatively characterized to be caffeoylglucaric acid isomers, in agreement with the profiles previously reported for *B. microphylla* G. Forst and *B. thunbergii* DC^46^. Compound **8** was tentatively identified as dihydrocaffeic acid 3-*O*-glucuronide with [M − H]^-^ *m*/*z* at 357.0826, and further confirmed from ions at *m*/*z* 181 due to loss of glucuronide from parent ion^45^.

*P. niruri* showed the presence of compound **10** for the first time, it showed a molecular ion peak [M + H]^−^ at *m/z* 623.0883 corresponding the molecular formula C_26_H_24_O_18_ with exact mass of 624.0963 and tentatively identified as dihydrovirganin. In addition, it showed the loss of C_9_H_8_O_8_ fragment at *m/z* 355.0665 [M–H–C_9_H_8_O_8_]^−^, and 234.0405 [M–H–C_9_H_8_O_8_–C_7_H_5_O_2_]^−^_._ The closely related virganin metabolite (C_26_H_22_O_18_) was identified in *P. niruri*^47^, *P. virgatus*^48^, and in *P. urinaria* and* P. amarus*^49^.

The metabolite **30** was tentatively characterized as elagitannin 4-*O*-brevifolincarbonyl- 1-*O*-galloyl- 3,6-*O*-hexahydroxydiphenoyl-*D*-glucopyranose^50^ with a molecular formula C_40_H_28_O_25_ and exact mass of *m*/*z* 908.0920. It showed the presence of [M–H]^−^ at *m/z* at 907.0847 and fragments of *m*/*z* at 611.0708, and 169.0141.

The metabolite **35** was tentatively identified as azelaic acid, a dicarboxylic acid had the molecular formula C_9_H_16_O_4_ and exact mass of 18.0974. Azelaic acid produced *m/z* 187.0974 [M–H]^−^ as the ion base peak in negative ion mode^51,52^. Azelaic acid is an organic acid compound observed in wheat, rice, rye and barley and it has antimicrobial and anti-inflammatory properties, which make it effective in the treatment of skin conditions like acne and rosacea^51^.

Compound **36** was observed only in negative ion mode and was identified as 3,4,5,7-Tetrahydroxyflavone-8-sulfonic acid (luteolin-8-sulfonic acid), a flavone sulfonic acid had the molecular formula of C_15_H_10_O_9_S and exact mass of 366.0046 and *m/z* 364.9973 [M–H]^−^. It showed mass ion *m/z* 285.0403 [M–H–SO_3_]^−^ corresponding to the loss of a sulfonyl group.

Peaks from **41** to **43** were identified as fatty acid isomers. They had deprotonated molecular ions [M–H]^−^ at *m/z* 327.2175, and fragments ions at *m*/*z* 285.0797, 215.1289, 171.1027, 85.0299. They were identified as trihydroxy-octadecadienoic acid isomers. On the other hand, the peaks from **44** to **47** were identified as fatty acid isomers of trihydroxy-octadecanoic acid. They had deprotonated molecular ions [M–H]^−^ at *m/z* 329.2332.

Six lignans were characterized in the current study, while only four were nirtetralin, phyllanthin, hypophyllanthin, and niranthin*,* were previously confirmed from our previous work^25^.

### Biological findings

Breast cancer continues to be a major global health problem and it possess high incidence rate in women with subsequent very high rates of morbidity and mortality^53,54^. Doxorubicin (DOX) is one of the mostly used chemotherapeutic medications for breast cancer. While DOX treatment initially causes clinical responses, its longstanding success may be halted due to the emergence of drug toxicities and resistance^55^. Currently, combining the natural products with chemotherapeutic agents has drawn researchers’ attention as it was observed to enhance the effectiveness of conventional chemotherapeutic medications and/or safeguard patients from their adverse effects^56^.

Therefore, in this study, we explored the potential cytotoxic activity of *P. niruri*, a well-established bioactive medicinal herb, as well as its potential influence on the cytotoxic profile of DOX in naïve (MCF-7) and doxorubicin-resistant (MCF-7^ADR^) breast cancer cells. The literature reported some in vivo and in vitro studies of anticancer activity of *P. niruri* in various types of malignancies^57^. To the best of our knowledge, this is the initial investigation for the impact of *P. niruri* on resistant breast cancer cells.

According to our data, all tested extracts, and fractions of *P. niruri* till did not show any promising cytotoxicity against the breast cancer cells alone under investigation (MCF-7 & MCF-7^ADR^) except for the methylene chloride fraction (**F-3**). It showed weak cytotoxicity against MCF-7 cells (IC_50_ = 97.2 μg/ml) and surprisingly higher activity against the DOX resistant breast cancer cells (MCF-7^ADR^). These findings were consistent with a few previous reports for the potential anticancer activity of aqueous and methanolic extracts of *P. niruri* against MCF-7 cells^20,58^.

However, the promising cytotoxicity of CH_2_Cl_2_ fraction against MCF-7^ADR^ cells may be in part due to the reported P-glycoprotein (Pgp) inhibitory activity of *Phyllanthus* extracts in Pgp overexpressing cell lines^59,60^. The activity of Pgp is implicated in multidrug resistance (MDR) of breast cancer and MCF-7^ADR^ cell line is the commonly used breast cancer cell model for the study of MDR relayed resistance^61,62^.

The discrepancy in the effectiveness of *P. niruri different *extracts against cancer cells may be attributed to the difference in the present of bioactive substances. Therefore, untargeted metabolite profiling of the MeOH extract and the active cytotoxic CH_2_Cl_2_ fraction was performed using LC-DAD-QToF. The LC–MS profiling of the active CH_2_Cl_2_ revealed the presence of main compounds such as the ellagitannin phyllanthussiin U (observed in positive mode) and flavonoids galangin-8-sulfonic acid and niruriflavone (marked in negative ion mode), in addition to six lignans nirtetralin, phyltetralin, phyllanthin, hypophyllanthin, niranthin and isolintetralin. These lignans are well known for their anticancer activities^63,64^ that enrich the active CH_2_Cl_2_ fraction as seen in LC–MS.

In MCF-7 cells, combining DOX with *P. niruri extracts* (except for CH_2_Cl_2_ fraction) at a sub-cytotoxic concentration unexpectedly resulted in an extensively negative modification of doxorubicin’s activity. These combinations not only didn’t show improvement in DOX-induced viability inhibition but additionally showed an increase in the IC_50_ values of DOX in combinations to nearly ten times of DOX alone, suggesting an antagonistic type of interaction. This makes a lot of sense if we consider the strong antioxidant properties (due to its phenolic content) of this fraction and the mode of action for DOX which involves the generation of reactive oxygen species. Also, this finding is in line with previous observations for the combinations that sometimes resulted in antagonistic effects suggesting that combination at different dose levels was diverse and specific to each combination and cell line^65^. The involved mechanisms in these interactions between DOX and *P. niruri* warrants further evaluation.

Surprisingly, in MCF-7^ADR^, the opposite occurred for most combinations with DOX, which showed potentiation of activity manifested by significant decrease in the IC_50_ values of DOX in combinations. These findings suggested that *P. nurini* derivatives could be effective MDR reversal agents, and accordingly they will synergize the activity of most of the standard chemotherapeutics such as DOX^66^. Another important observation, CH_2_Cl_2_ fraction showed the maximal DOX activity potentiation in both cell lines.

According to our observation, *P. nurini* CH_2_Cl_2_ fraction alone showed moderate anti-proliferative effects. However, it enhanced the cytotoxic profile of DOX by 4 folds and 40 folds against MCF-7 and MCF-7^ADR^, respectively. Several studies described the significance of *Phyllanthus* alone as an anti-cancer agent against distinct types of malignancy^20,22^. In addition, several publications including ours showed promising chemomodulatory activities of *Phyllanthus* to several chemotherapeutics in various types of malignancy^65,67^.

Combining the results of the two cell lines, Fr-3 had the lowest IC_50_ values against naïve and resistant MCF-7 cells. Thus, it was selected for further TLC fractionation and investigation for their cytotoxicity and chemomodulatory potential against breast cancer cells. In the same manner, subfractions behave, showing more cytotoxic activity as well as chemomodulatory effect against MCF-7^ADR^. In addition, TLC fractionation showed that **B2** subfraction (*R*_f_ 0.2–0.35) was the most potent one in terms of cytotoxicity when combined with DOX.

Some studies suggested that the production of reactive oxygen species (ROS) after DOX treatment is responsible for the cytotoxicity in cancer cells, which could stimulate the production of antioxidant enzymes, like GSH, as a protective defense mechanism of cancer cells making it resistant to oxidative damage. Similarly, greater sensitivity to DOX was observed in breast cancer when the GSH levels decreased^68,69^. Herein, GSH level reduction observed by *Phyllanthus niruri* may explain the enhanced chemotherapeutic effect of DOX via sensitizing the resistant MCF-7^ADR^ cells^70^.

This finding supports our hypothesis that combinations of plant extracts and chemotherapeutic agents may allow for a significant reduction in the dosage of the more toxic chemotherapeutic agent while retaining the therapeutic efficacy and minimizing toxicities not only in naïve tumor cells but also and to a greater extent in the resistant tumor cells. Therefore, additional studies are needed for separation of pure compounds and further elucidation of mechanistic studies for the anticancer and chemomodulatory effects in naïve as well as resistant breast cancer cells.

## Conclusion

Combined pharmacotherapy is a common approach that would improve the anticancer activities of cytotoxic drugs while lowering chemo-resistance of cancer cells. Accordingly, and to the best of our knowledge, this is the first study considering anticancer properties of *P. niruri* in resistant breast cancer alone and in combination with DOX. *P. niruri* is a promising chemomodulatory agent against resistant breast cancer. Hence, future investigations should include its effect in detail on resistant breast cancer and other MDR overexpressing cancer cells.

## Supplementary Information

Supplementary Information.

## Data Availability

All data generated or analyzed during this study are included in this published article and its supplementary information file.
