# Supplementary material for: Cytotoxic and chemomodulatory effects of Phyllanthus niruri in MCF-7 and MCF-7ADR breast cancer cells
Source: Sci Rep. 2023 Feb 15;13:2683. doi: 10.1038/s41598-023-29566-0 (PMC9932073; doi:10.1038/s41598-023-29566-0)
Supplement: Supplementary file 1 — Supplementary Information. [file 41598_2023_29566_MOESM1_ESM.pdf]

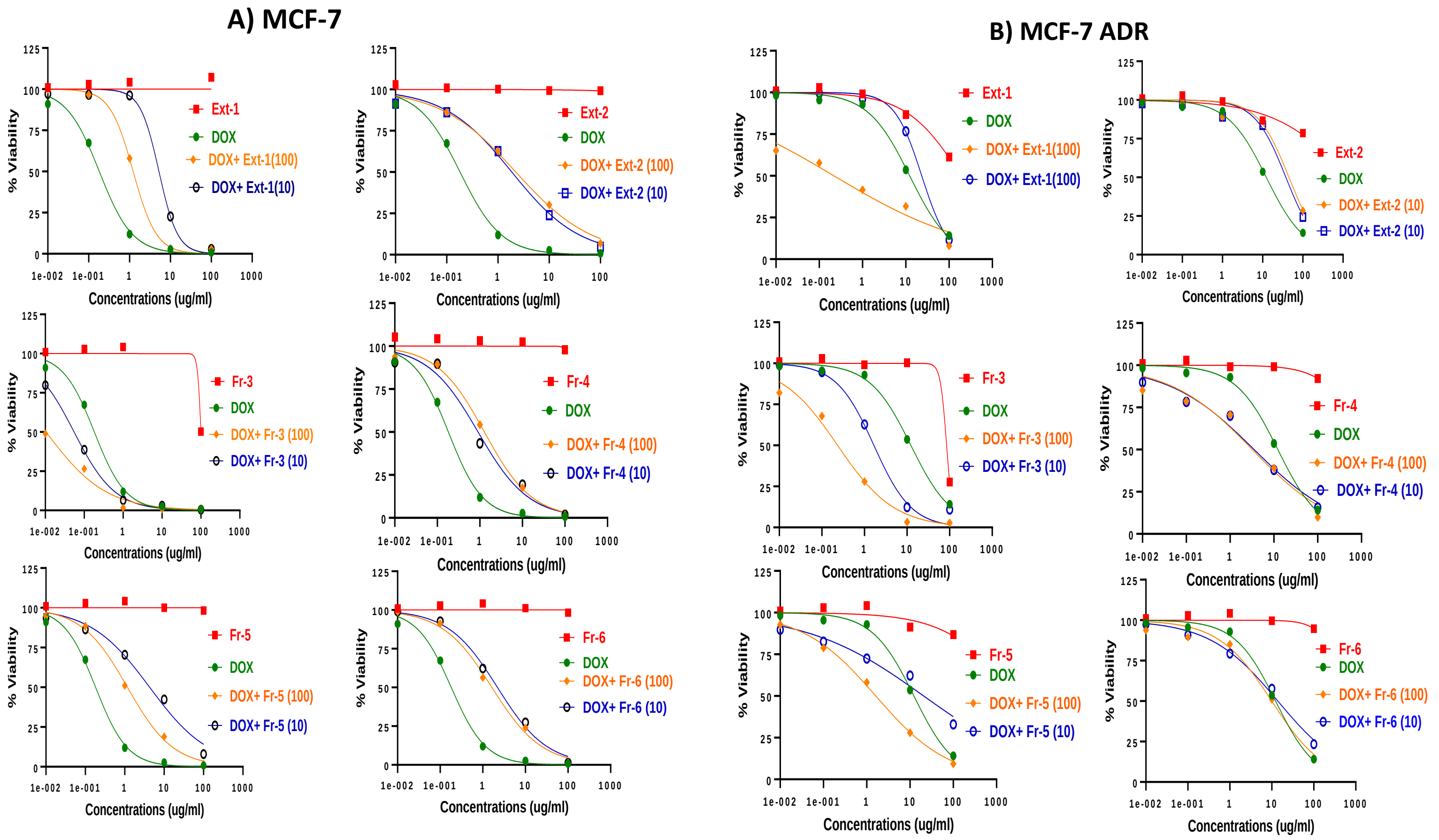

Figure 1S. The effect of six extracts and fractions of *Phyllanthus niruri* on the cytotoxicity of DOX in MCF-7 (**A**) and MCF-7^{ADR} (**B**) cell lines. Cells were treated with serial dilution of DOX, extracts or their combinations for 72 h. Cell viability was evaluated using SRB assay.

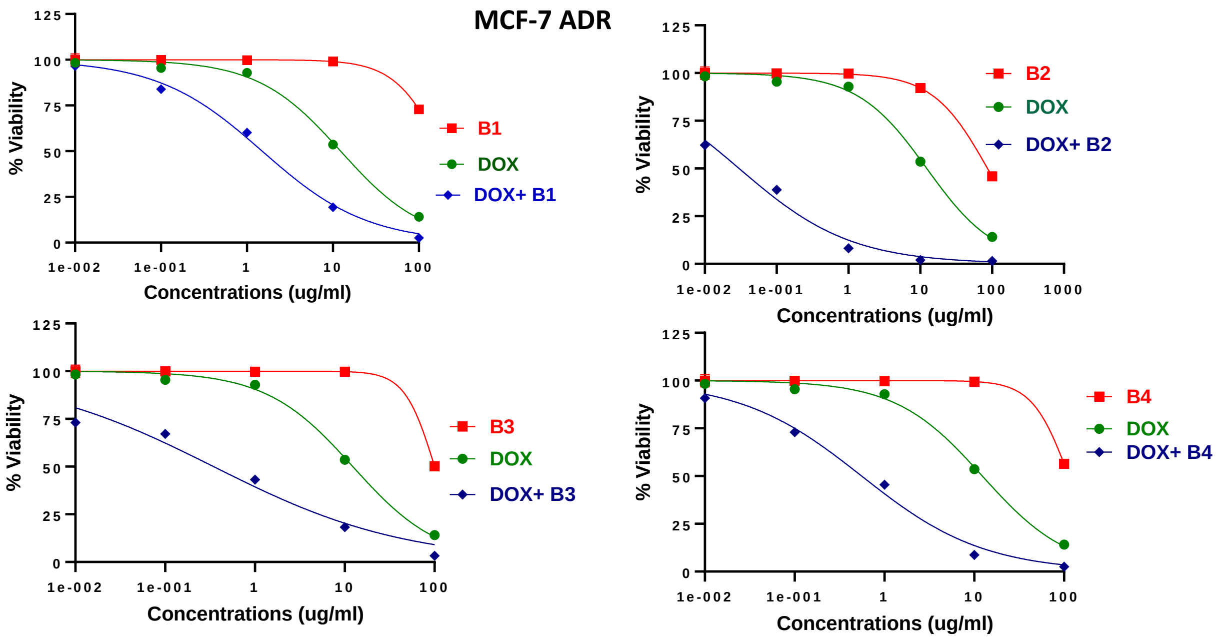

Figure 2S. The effect of four subfractions of Fr-3 fractions of *Phyllanthus niruri* on the cytotoxicity parameters of DOX in MCF-7^{ADR} cell lines. Cells were treated with serial dilution of DOX, subfractions or their equitoxic combinations for 72 h. Cell viability was evaluated using SRB assay.

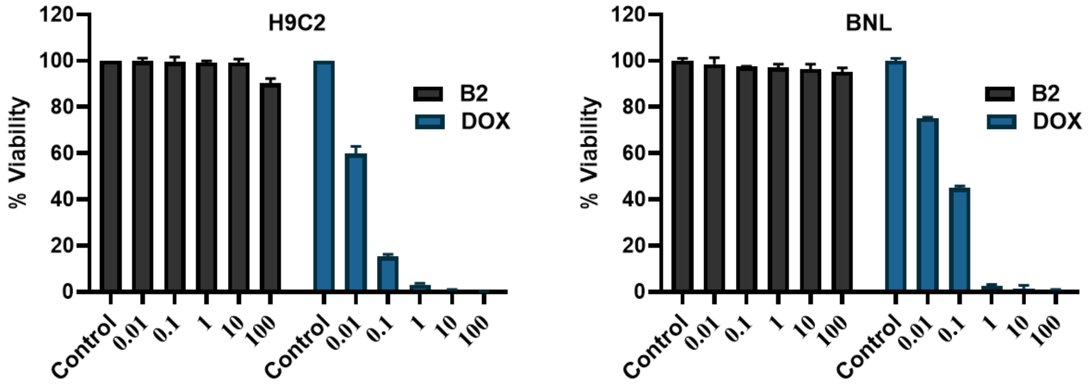

Figure 3S: The effect of the most potent fraction (B2) of *Phyllanthus niruri* on the viability of H9C2 and BNL cells. Cells were treated with serial dilution of B2 and DOX (0.01, 0.1, 1, 10, and 100 ug/ml) for 72 h. Cell viability was evaluated using SRB assay.
